# Evidence of validity and reliability of the environmental action scale in Peruvian university students

**DOI:** 10.3389/fpsyg.2023.1232397

**Published:** 2023-11-22

**Authors:** Antonio Serpa-Barrientos, Enrique Giovanni Pérez-Flores, Gerardo Manuel Bellido-Figueroa, Jacksaint Saintila

**Affiliations:** ^1^Departmento de Psicología, Universidad Nacional Mayor de San Marcos, Lima, Peru; ^2^Department de Psicología, Universidad César Vallejo, Lima, Peru; ^3^Escuela de Medicina Humana, Universidad Peruana Unión, Lima, Peru

**Keywords:** environmental action, validity, reliability, psychometric properties, university students

## Abstract

**Background:**

The environmental action scale is used to measure the degree of participation in collective environmental actions and has been shown to have adequate psychometric properties in developed countries. However, there are still no studies that have evaluated its performance in the Peruvian population.

**Methods:**

In this instrumental study, the environmental action scale (EAS) was translated, adapted, and validated. The EAS was administered to 352 university students between 18 and 35 years of age (*M*_age_ = 23.37, *SD* = 2.57) from different cities in Peru. A validity analysis was performed using two sources of evidence: content validity and internal structure, carrying out an exploratory factor analysis (EFA) and a confirmatory factor analysis (CFA).

**Results:**

The structure of the scale has been organized into three oblique factors. The findings confirmed the reliability and validity of the three dimensions of the EAS.

**Conclusion:**

Therefore, this scale is considered a valid option for assessing environmental action.

## Introduction

Despite the growing concern about the deterioration of the environment and biodiversity, as well as for the negative effects of human actions on various species and their ecosystems, this issue has not yet been given due importance in the policies of many countries (Carmona et al., 2023). On the other hand, although several groups or movements have emerged in recent decades to advocate for environmental protection and conservation (Mostafavi et al., 2022), it has not yet been possible to create a generalized awareness of the importance of changing life habits to reduce environmental impact (Muhammad et al., 2020). Therefore, the environmental impact of human activity has led the planet to its sixth mass extinction of species (Bermúdez-Tamayo et al., 2023). However, during 2020, in response to the appearance of SARS-Cov-2, the governments of different countries implemented social immobilization measures that lasted for several months (WHO, 2020). These measures had a significant effect on reducing pollution globally (Muhammad et al., 2020) which demonstrated that large-scale community action can bring about positive environmental change (Pérez-Vásquez and Arroyo-Tirado, 2022).

According to the United Nations (UN, 2018), the unregulated exploitation of natural resources has been one of the main causes of armed conflicts that have occurred worldwide since the 1960s, this has resulted in the extinction of some resources and the degradation of the environment, which, in turn, affects people’s well-being (Razo, 2021). Because human beings depend on the environment for their daily actions, these can aggravate environmental problems, since sometimes ecosystems are destroyed to satisfy survival needs, which can lead to environmental degradation (Majeed and Ozturk, 2020; Sahani et al., 2022). In fact, the increase in global temperature over the last decade is a direct consequence of ecosystem degradation (Weiskopf et al., 2020). Consequently, the climatic impact could be irreversible and generate global consequences, such as sea level rise, greenhouse effect, melting of the poles, among others (Kim et al., 2014; Razo, 2021; Rocque et al., 2021). According to estimates, over the next two decades, it is expected that the environment will suffer serious consequences due to a significant increase in temperature (UN, 2020). This increase is due to the high emission of harmful substances that cause irreparable damage to the atmosphere. Consequently, new diseases and genetic changes may appear in humans as well as in other species (Kim et al., 2014; Rocque et al., 2021).

At the national level, it has been observed that climate change has had a negative impact on the ecosystems of the Peruvian Amazon region. This is due to informal mining activity that has caused high levels of water contamination with mercury (MINAM, 2020). This situation has generated environmental problems that have affected the health and social well-being of the population (Schmeller et al., 2020). In addition to water contamination, the Ministry of Environment has reported that, during the pandemic restrictive measures, illegal deforestation of 7,119 hectares of Amazon rainforest has been recorded, which represents a worrying environmental situation (MINAM, 2020). It is fundamental to consider that Peru has a wide diversity in terms of climate, topography, and ecosystems, covering from the arid coast to the dense Amazon jungle and the Andes mountains (Peña et al., 2021).

Therefore, it is crucial to have scientific theories that explain people’s distant behavior towards the ecosystem. Additionally, validated measurement instruments are necessary to assess the level of environmental awareness in the Peruvian context and understand the magnitude of the phenomenon. In fact, there are currently instruments that assess the level of environmental actions, such as the Delaware Environmental Consciousness Scale (ECA_FMEP) (Laso et al., 2019a). This instrument addresses the measurement of environmental consciousness in specific contexts of initial education. Similarly, the Environmental Values Scale (2-MEV) (Bogner, 2018) focuses on secondary school students, as the author seeks to obtain an adolescent perspective. It has been proposed that adolescents have a more optimistic view of the world.

While these scales evaluate individuals’ willingness to prioritize environmental protection at an individual level, they do not specifically consider collective action. Therefore, there was a need to adapt the EAS scale to the national context since there is a lack of psychometric adaptation studies on this variable in Peru, which is aimed at a general audience (CONCYTEC, 2021). The scale analyzes the commitment to civic actions aimed at producing an environmental impact, based on its three dimensions: Environmental Citizenship Action, Environmental Education, and Environmental Activism. In this sense, the adaptation of the EAS scale will allow for a more comprehensive and precise assessment of individuals’ commitment to activities that promote environmental protection and conservation.

In fact, measuring levels of environmental consciousness from a psychological perspective will help us understand the psychological processes that influence how people interact with the environment, as well as their perception of the severity of environmental issues and the personal values that influence such behaviors (Laso et al., 2019a; Garrido et al., 2022). By understanding these factors, we can gain a clearer perspective on the population’s level of commitment towards ecosystem conservation. It is important to highlight that the scale promotes civic action and the extrinsic knowledge acquired through society in which the individual develops. This generates an interest in preserving the environment, leading to more responsible behaviors and an activist attitude towards environmental care. This, in turn, will enable the development and implementation of strategies that foster environmental awareness and protection, promoting sustainable behaviors in the long term (Pérez and Medrano, 2010).

In a study conducted by Carmona-Moya et al. (2019) on a Spanish sample, through the analysis of the internal structure of the Academic Behavior Self-Efficacy Scale (EACA), it was demonstrated that the assessment is consistent with two aspects of the construct: “Participatory Activities” and “Leadership.” Additionally, it was found that this assessment is related to factors such as “Environmental Identity” and “Moral Beliefs.”

Similarly, Laso et al. (2019a) designed and validated a scale to measure environmental consciousness in a sample from Spain. The scale consists of four dimensions (affective, cognitive, conative, and active) that assess environmental consciousness in specific contexts. The results of the confirmatory factor analysis supported the adequate validity of the instrument. The Environmental Action Scale (EAS) designed by Alisat and Riemer (2015) aims to measure collective consciousness and the values associated with preserving a healthy ecosystem. This scale is based on the theory of significant environmental behavior proposed by Stern (2000). According to this theory, intense and highly committed activist behaviors in the public sphere are referred to as leadership actions, while less committed activist behaviors indicate little interest in environmental protection and care. Consequently, it is recommended to promote commitments that benefit the environment from the communities involved. This measure aligns with the importance of promoting collective change and strengthening citizen commitment to environmental protection, as proposed in Stern’s theory of significant environmental behavior (Stern, 2000).

Based on the above, the objective of this research was to translate, adapt, and validate the EAS scale in the Peruvian context, evaluating its content-based validity, internal structure, and reliability through internal consistency, as well as to evaluate whether the model is equivalent according to sex.

## Materials and methods

### Participants

A total of 378 Peruvian university students were evaluated, of which 26 records were eliminated because they did not meet the established minimum age (≥18 years), leaving a total of 352 evaluated for the corresponding analysis. Consequently, the characteristics of the final sample are as follows: university students aged between 18 and 35 (M_age_ = 23.37, *SD* = 2.571), of whom 38% were male and 62% female. The sample included participants from various cities in Peru, with Piura being the most represented city (62%), followed by Chiclayo (7%), Lima (7%), Tumbes (6%), and other cities (18%) (Table 1).

**Table 1:** Demographic description of participants (*n* = 352).

	Categories	*f*	%
Gender	Male	135	38
	Female	217	62
Age	18–20	99	28
	21–25	197	56
	26–30	35	10
	31–35	21	6
Cities	Piura	217	62
	Chiclayo	26	7
	Lima	26	7
	Tumbes	22	6
	Trujillo	17	5
	Arequipa	10	3
	Cajamarca	10	3
	Huánuco	10	3
	Cusco	8	2
	Rioja	6	2

*f*, frequency.

### Instruments

The Environmental Action Scale (EAS) (Alisat and Riemer, 2015) consists of 18 items with responses scored on a range from never (0), indicating that the evaluated action or attitude never occurs, to always (4), indicating that it occurs on all relevant occasions. Additionally, the instrument comprises three dimensions: (1) “Environmental Citizen Action,” which refers to the individual and collective actions that people take in their daily lives to contribute to the protection and conservation of the environment, (2) “Environmental Education,” which focuses on individuals’ knowledge and environmental awareness, evaluating their understanding of environmental issues and their willingness to learn and disseminate information related to the environment, and (3) “Environmental Activism,” which refers to active participation in movements and collective actions aimed at promoting environmental changes and policies. This can include involvement in awareness campaigns, conservation activities, and the promotion of sustainable policies. The instrument demonstrates high reliability values, with *α* = 0.92 and total item agreement ranging from 0.43 to 0.80.

### Procedure

The project was approved by a Research Ethics Committee (Registration Code: N°DR-0023-P-22), and authorization was obtained to adapt the EAS scale to the Peruvian context. The information was collected through a virtual form designed by the researchers using Google Forms and disseminated through social networks and emails. The form included the research objectives, electronic informed consent, confidentiality of the results and anonymity of the participants, as well as sociodemographic data. Informed consent was obtained from all participants. The data were collected between August 20th and September 20th, 2022, and subsequently, statistical analysis was conducted.

### Statistical analysis

After administering the EAS, we proceeded to systematize the data in Microsoft Excel version 2016 for validation. Subsequently, we imported the data into the statistical software SPSS v26, where we conducted a series of analyzes aimed at verifying assumptions related to data normality. Firstly, we examined univariate normality assumptions based on indicators such as skewness and kurtosis, which were expected to fall within the range of ±1.5 (Pérez and Medrano, 2010). Furthermore, we calculated Z-score values for each item and considered items with values outside the ±3.0 threshold as outliers (Tabachnick and Fidell, 2019). To detect multivariate outliers, we employed the Mahalanobis distance and considered a value as an outlier if it showed a significance level in the chi-square test of less than 0.001 (Mahalanobis, 2019).

Additionally, we calculated the Mardia’s coefficient of kurtosis (Mardia, 1970) to assess multivariate normality, aiming for a critical ratio equal to or less than 5.0 (Yuan et al., 2005). These analyzes were conducted with the purpose of ensuring data validity and facilitating the precise selection of the most suitable estimator. In situations where the assumption of a multivariate normal distribution was not met, we chose to use the weighted least squares means and variance-adjusted (WLSMV) estimator (Muthén, 1984; Muthén et al., 1997). Conversely, in cases where the data’s ordinal nature prevailed, we applied the maximum likelihood (ML) estimator.

To evaluate the internal structure, we performed a confirmatory factor analysis (CFA) using R Studio software, particularly the Lavaan package (Rosseel, 2012). Furthermore, we considered factor loadings equal to or greater than 0.40 as adequate (Brown, 2015).

Regarding fit indices, we deemed *X*^2^/df ratio values acceptable when they fell between 2 and 3, with a maximum value of 5, as established in previous research (Iacobucci, 2010; Escobedo et al., 2016). Similarly, we considered it appropriate for the CFI and TLI parameters to exceed 0.95, as proposed in previous studies (Hu and Bentler, 2009), and for the SRMR and RMSEA values to be equal to or less than 0.05, which is considered acceptable (Browne and Cudeck, 1992). Since the CFA results did not meet the recommended fit criteria and with the aim of finding theoretical correspondence and identifying new factors, we decided to conduct an Exploratory Factor Analysis (EFA). To assess the instrument’s reliability from a dimensional perspective, we calculated alpha (α) and omega (ω) coefficients, considering values equal to or greater than 0.70 as appropriate, following previous recommendations (Campo-Arias and Oviedo, 2008).

To assess whether the EAS is comparable according to gender, a step-by-step analysis of each model was carried out, applying constraints in succession. It started with the configural constraint, followed by the Threshold, metric, scalar and, finally, the strict constraint. This process was performed using a multigroup CFA (Chen, 2007; Byrne, 2008). For this purpose, the following guidelines were considered: ΔCFI ≤0.01 and ΔRMSEA ≤0.015. These criteria facilitated the evaluation of the parameters derived from each of the constraints imposed in the model (Cheung and Rensvold, 2002).

## Results

### Translation and adaptation of the EAS

After completing the translation of the EAS, a linguistic adaptation was carried out by six psychologists specialized in Environmental Psychology, where they suggested providing more details and explanations in items 5, 6, 12, 13, 14, 15, 16, and 17 for a better understanding and clarity. Following this, the results were analyzed using Aiken’s V coefficient, where the results were between 0.78 and 1.00. as appropriate (Charter, 2003; Pérez and Medrano, 2010; Ledesma et al., 2019). Linguistic adaptation of the Environmental Action Scale (EAS) can be found in Supplementary Table S1.

### Verification of typical scores

After justifying the validity of the content with experts and systematizing all information, we started the statistical analysis. In addition, the skewness and kurtosis coefficients were obtained for each of the items, the results showed that item 2 presented the minimum value of skewness (−0.262), while item 12 presented the maximum value (1.823). Regarding kurtosis, item 3 presents the minimum value (−1.213) and item 12 the maximum value (2.41), which indicates that the values of the latter item are above the recommended value ±1.5 (Pérez and Medrano, 2010). Continuing with the univariate normality analysis, it was also evaluated through the calculation of Z-scores for each item, the results allowed describing that most of the items scored within the expected range of ±3.0 except for items 2, 4, 7, and 13, since the values will be obtained outside the established range (Kapoor, 2016). These results provide additional information to the analysis of skewness and kurtosis, complementing with the verification that the data do not follow a univariate distribution in all cases (all 18 items), putting an early warning to be more careful on these items when evaluating the internal structure by means of the CFA. Furthermore, Mardia’s coefficient criteria were used to obtain multivariate normality values (as an appropriate value G2 ≤ 5.0), which indicated that the data do not follow a multivariate distribution (G2 = 17.852). Therefore, the robust method (WLSMV) was used to obtain the adjustment parameters using the CFA. In addition, because the data are ordinal and do not have a proper multivariate normal distribution, the use of the robust estimator is justified.

### Confirmatory factor analysis

The model was analyzed by calculating the goodness-of-fit measures, according to the original model (M1, see Table 2), in which the following values were obtained: X^^2^/df = 6.278, CFI = 0.917, TLI = 0.906, and SRMR = 0.083, RMSEA = 0.123. Consequently, these parameters are not acceptable for X^^2^/df (< 5.0) (Wheaton et al., 1977). Likewise, the SRMR and RMSEA parameters obtained values outside the acceptable ranges; however, the fit indices such as CFI and TLI were greater than 0.90, considered acceptable, but not optimal. Consequently, the empirical model would not be fitting the hypothetical model. Additionally, the interfactor correlation was quite high (φ = 0.90), whose interpretation allows us to suggest that the factors would have a redundant (collinear) behavior, i.e., it would not be completely clear that the construct “Environmental action” has two dimensions, but rather a unidimensional behavior. In this sense, it was necessary to evaluate a second model (M2, see Table 2) to explain whether the construct could be plotted in a unidimensional way, while the fit index parameters obtained from the unifactorial model did not support the unidimensional proposal either, because the fit indexes were not appropriate (X^^2^/df = 6.04, CFI = 0.805, TLI = 0.779 and SRMR = 0.072, RMSEA = 0.120). Consequently, the evaluation of the internal structure through the CFA of “Environmental Action” in this sample of Peruvian university students was not satisfactorily evaluated under an oblique model or in a unidimensional manner. Moreover, the index modification (IM) parameters were numerous (more than 100 IM) for each model evaluated. Therefore, the psychometric recommendation suggests that the factorial structure be evaluated using the EFA technique, to identify the new structural configuration of the construct.

**Table 2:** Fit index of the proposed models using CFA.

	*χ* ^2^	df	*χ*^2^/df	CFI	TLI	SRMR	RMSEA	RMSEA 90% IC	
								Lower	Upper
M1	841.22	134	6.278	0.917	0.906	0.083	0.123	0.115	0.131
M2	815.40	135	6.040	0.805	0.779	0.072	0.120	0.112	0.128

*χ*^2^, Chi-square; df, degree of freedom; CFI, Comparative Fit Index; TLI, Tucker Lewis index; SRMR, root mean square standardized residual root; RMSEA, root mean square error of approximation.

### Exploratory factor analysis

**Figure 1:**
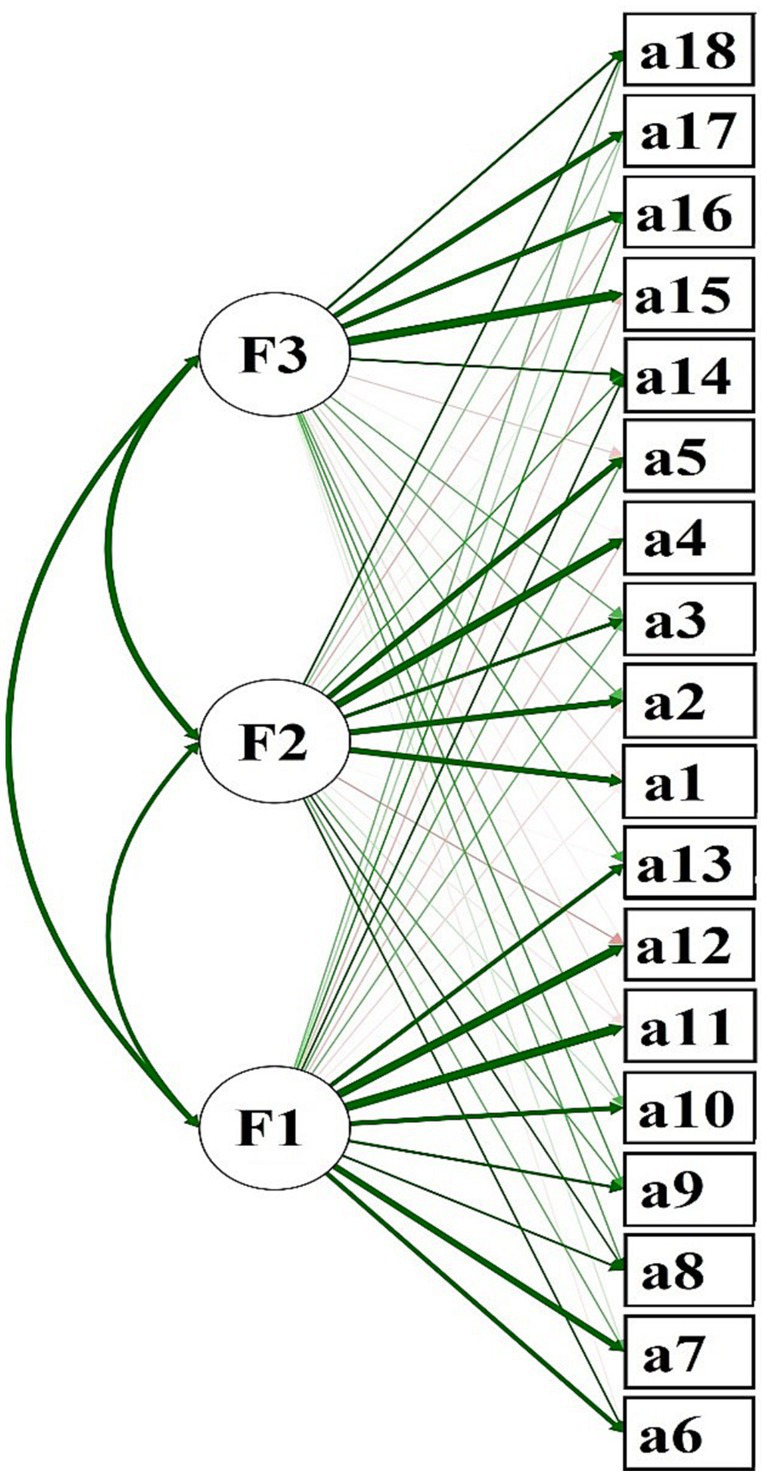
Graphical representation of the EFA. F1: Environmental citizen action, F2: Environmental education, F3: Environmental activism. The green arrow indicates positive association, while the red arrows indicate negative association. The green arrows with greater thickness indicate that the association between the factor and the items is of greater magnitude (factorial loading), the figure obtained with the JASP Teem software.

Indeed, after evaluating two structural models with unreliable estimates, it was considered necessary to explore the construct by means of the EFA technique, to identify new possible factors that could conglomerate the items to support the new conformation of the construct. In this sense and under the psychometric recommendations (as the number of index modifications was higher than 100 and the evaluation of the fit indexes of the previous models were not as expected), we proceeded to evaluate. The results were quite promising, where the sample adequacy through the KMO was greater than 0.80 (KMO =. 935) considered as good, the Bartlett’s Sphericity parameters allow to describe that the correlation matrix an image (χ^2^ = 3,562 df = 153, *p* < 0.001); Consequently, these parameters justified the correct execution of the exploratory factorial technique (see Table 3 and Figure 1). As for the factor loadings of the EFA, for each factor identified, they were above the recommended threshold (*λ* ≥ 0.30) and the values of communality and interfactor correlations were quite appropriate (*h*^2^ ≥ 0.40; see Table 3).

**Table 3:** Factor loadings EFA.

	Factors			*h* ^2^
	Environmental citizen action	Environmental education	Environmental activism	
Item11	***0.86***	−0.04	−0.04	0.68
Item12	***0.79***	−0.09	−0.05	0.53
Item7	***0.68***	0.15	0.03	0.61
Item10	***0.56***	0.06	0.21	0.53
Item6	***0.52***	0.29	0.01	0.50
Item13	***0.48***	0.00	0.22	0.40
Item9	***0.41***	0.19	0.26	0.52
Item8	***0.32***	0.27	0.24	0.48
Item4	−0.06	***0.76***	−0.03	0.51
Item2	−0.05	***0.71***	0.16	0.63
Item1	−0.02	***0.68***	−0.07	0.40
Item5	0.17	***0.63***	−0.06	0.48
Item3	0.15	***0.50***	0.19	0.54
Item15	−0.07	0.00	***0.94***	0.81
Item17	0.07	0.14	***0.62***	0.58
Item16	0.28	−0.07	***0.60***	0.57
Item18	0.16	0.32	***0.40***	0.56
Item14	0.32	0.25	***0.37***	0.63
% of Variance	21.34	17.72	16.24	
Cumulative %	55.29			
F1	—	0.477	0.556	
F2		—	0.625	
F3			—	
Bartlett’s Test of Sphericity	*χ*^2^ = 3,562, df = 153, *p* < 0.001			
KMO	0.935			

“Minimum residual” extraction method was used in combination with a “oblimin” rotation. Those in bold italics are items with acceptable factor loadings.

After the evaluation of the construct by means of the EFA, the percentage of variance obtained is as follows: The proportion of variance explained by the *first factor* (named “Environmental citizen action,” after the syntactic analysis of each of the items) composed of items 6, 7, 8, 9, 10, 11, 12, and 13 explained 21.34% of the total variance of the construct. The *second factor* whose items are 1, 2, 3, 4, and 5 (called “Environmental education”) explains up to 17.72% of variance and the *third factor* whose items are 14, 15, 16, 17, and 18 (called “Environmental activism”) explains up to 16.24% of variance. In summary, the total variance explained on the construct by the EAS is up to 55.29%. That is, these described parameters allow us to justify that the environmental action construct, measured through the EAS, meets the evidence of validity based on the internal structure by means of the EFA (see Table 3).

### Reliability of the EAS

After that, the reliability was evaluated by internal consistency analysis using the Alpha and Omega coefficients. This analysis was performed for each of the dimensions of the environmental action scale, obtaining appropriate values for the dimensions “Environmental Citizen Action” (*ω* = 88 and *α* = 0.88), “Environmental Education” (*ω* = 0.83 and *α* = 0.82), and “Environmental Activism” (*ω* = 0.87 and *α* = 0.87) (Table 4). In summary, these parameters allow us to justify that the EAS, evaluating by its internal structure using the EFA, presents good internal consistency (*α* > 0.70), justifying the reliability of the instrument.

**Table 4:** Internal consistency reliability statistics.

	Items	M	SD	r_itc_	*α*	*ω*
Environmental citizen action	a6	1.477	1.27	0.641	0.882	0.887
	a7	0.813	1.2	0.737		
	a8	1.315	1.47	0.594		
	a9	1.372	1.29	0.669		
	a10	1.25	1.32	0.682		
	a11	0.744	1.14	0.724		
	a12	0.588	1.04	0.621		
	a13	1.142	1.31	0.561		
Environmental education	a1	2.91	0.867	0.558	0.822	0.830
	a2	2.37	1.186	0.715		
	a3	1.84	1.376	0.63		
	a4	2.88	1.027	0.609		
	a5	2.15	1.308	0.615		
Environmental activism	a14	1.45	1.3	0.707	0.876	0.877
	a15	2.07	1.3	0.771		
	a16	1.43	1.33	0.672		
	a17	2.14	1.25	0.702		
	a18	1.7	1.32	0.674		

M, Mean; SD, standard deviation; r_itc_, total corrected item ratio.

Measurement invariance analysis was carried out with the purpose of determining whether the structure and parameters of a measurement scale are consistent between different groups. In the context of this study, the comparison focused on discerning these equivalences between men and women. The results presented in Table 5 show the parameters derived from each constraint imposed: configural, Threshold, metric, scalar and strict, in that specific order. The information obtained indicates that the structure of the model is equivalent between genders. The differences detected for each constraint and in each fit index turned out to be statistically non-significant (Δ < 0.01), according to Cheung and Rensvold (2002). This suggests that it is feasible to compare the characteristics of environmental behavior among university students, taking gender into account.

**Table 5:** Measurement invariance analysis of the EAS scale according to sex.

Restriction	*χ* ^2^	Δ*X*^2^	Df	ΔDf	RMSEA	ΔRMSEA	CFI	ΔCFI	TLI	ΔTLI
Configural	462.71		264		0.092		0.959		0.953	
Threshold	476.74	38.564	299	35	0.087	−0.004	0.958	−0.001	0.957	0.004
Metric	490.58	13.875	314	15	0.083	−0.004	0.960	0.002	0.961	0.004
Scalar	502.78	11.250	329	15	0.078	−0.005	0.964	0.003	0.966	0.005
Strict	586.25	40.018	347	18	0.073	−0.005	0.966	0.003	0.970	0.004

*χ*^2^ Chi-square; Δ, parameter difference.

## Discussion

Environmental degradation and its impact on climate change may be one of the most significant concerns for humanity. For this reason, world leaders, businesses, and citizens gather each year at the Conference of the Parties (COP), organized by the United Nations Environment Agency (UN), to address this critical issue. The fundamental objective of the COP is to continue exploring recent advances in climate science, which will allow us to understand the phenomenon and its implications that have been generating global concern. In this sense, from the perspective of environmental psychology, the diverse behavioral implications of people and their close relationship with the environment have been investigated. Consequently, there is an evident need to understand the levels of environmental awareness using various instruments that can measure this variable from an environmental psychological perspective, which focuses on understanding the psychological processes that influence the way in which people interact with the environment, in addition to the perception of the seriousness of environmental problems and the personal values that influence these behaviors (Laso et al., 2019b; Garrido et al., 2022). When these factors are assessed using a measurement tool, a more accurate understanding of the population’s level of awareness of environmental care can be obtained (Wheaton et al., 1977). Therefore, strategies can be developed and implemented to foster environmental awareness and protection of the environment collectively, promoting long-term sustainable behaviors (Pérez-Vásquez and Arroyo-Tirado, 2022).

Consequently, the objective of this research was to adapt and validate the EAS in a sample of Peruvian university students. For this purpose, it is necessary to employ factorial techniques that can clarify the nature of the variable in the national context and its subsequent use. The internal structure of the EAS was successfully adapted and validated through the EFA; in addition, its reliability was determined by means of internal consistency in the study sample.

The results of the evaluation of the original two-factor model (“Participatory actions” and “Leadership actions”), proposed by Alisat and Riemer (2015), were not the most appropriate because the obtained values of the fit indices prevented corroborating the proximity of the empirical matrix to the hypothesized matrix, despite the fact that the values of the factor loadings were appropriate (λ ≥ 0.50). This controversy is probably due to aspects of the culture and idiosyncrasy of individuals who belong to each geographical context, because the perception and interpretation of the environmental phenomenon is influenced by the social and cultural factors of each region. Therefore, it is necessary to adapt this instrument, considering several factors or variables that could condition the understanding and comprehension of each of the items (Muñiz et al., 2013).

Consequently, the description of the parameters obtained from the two-factor oblique model [M1, proposed by Alisat and Riemer (2015)] and evaluated by Carmona-Moya et al. (2019) were inconclusive [interfactor correlation ≥0.85 (collinearity between factors) and fit indices with values below or above the established threshold], i.e., the construct would not be manifesting itself through two dimensions (“Participatory actions” and “Leadership actions”), but in a unidimensional way. Then, considering these observations, we proceeded to evaluate the internal structure of the “Environmental action” model for the second time in a unifactorial manner, obtaining unsatisfactory results, since the adjustment parameters also failed to justify that the construct is verified in a unidimensional manner. In addition to this, several MI greater than 100 possibilities was observed; this result allowed inferring that the structure of the EAS must necessarily be reviewed unsings an exploratory approach to identify new factors that would be grouping the items under a structure different from the one proposed by Alisat and Riemer (2015). Therefore, the internal structure was evaluated using the EFA.

The results of this new analysis using the EFA were the most appropriate in comparison to the results obtained using the CFA, showing that the construct is being represented by these three factors: “Environmental Citizen Action (ECA),” “Environmental Education (EE),” and “Environmental Activism (EA).” The naming of each factor followed a syntactic analysis of each of the items clustered in a specific factor to be renamed, for example: item 10 of the ECA dimension *(“Participé en una protesta o manifestación sobre un tema medioambiental”* / *“I took part in a protest/rally about an environmental issue”)*, item 2 of dimension EE *(*“*Participé en una actividad educativa (e.g., un taller) relacionado con el medio ambiente*”/ *Participated in an educational event (e.g., workshop) related to the environment”)*, and item 16 of the EA dimension *(*“*Organicé una actividad comunitaria (por ejemplo: limpieza de las calles, parques, ríos, canales, playas, entre otros)*” / “*Organized a community event which focused on environmental awareness”)*. Regarding the reliability of the EAS scale, specifically the three-factor oblique model, the values obtained allow us to affirm that the instrument has satisfactory parameters (alpha and omega coefficients), which suggests that the precision and accuracy with which the instrument captures the information is justified.

Additionally, the assessment of measurement invariance with respect to gender in this study is noteworthy. The derived parameters indicate that the model is invariant between genders, which facilitates the analysis of possible differences in the levels of environmental action among Peruvian university students according to their sex. It is relevant to mention that previous research did not present evidence of invariance analysis, which makes this finding a valuable contribution for future work in the area.

### Limitations

The results obtained from this research should be interpreted considering some limitations. Firstly, the measurement invariance, which is essential for comparing groups and determining if the interpretation of the phenomenon differs, such as based on gender, has not been assessed. Additionally, it is important to evaluate longitudinal invariance to ensure the stability of the instrument over time when implementing intervention programs (Brown, 2015). Secondly, the sample size was determined based on participant availability and characteristics, using non-probability sampling. However, this could pose a risk to the external validity of the study, as the standardization of the sample to the population is a crucial objective in quantitative research (Ato et al., 2013). For future research, it is recommended to increase the sample size to reduce error variability. Thirdly, it is relevant to note that the lack of convergent validity limits the interpretation and generalizability of the results. Therefore, it is advisable to include additional measures that allow evaluating the relationship between the variables of interest and other related variables within the study’s context. This inclusion will strengthen the validity of the findings and provide a more comprehensive understanding of the phenomena studied in different situations. Lastly, the evaluation of item stability using the test–retest method has not been conducted, which could enhance the reliability of the EAS scale. Hence, it is suggested that future research includes this as a research objective.

## Conclusion

It can be concluded that the EAS scale has adequate psychometric properties that support the interpretations made. Therefore, this scale is considered a valid option for use in research in the field of environmental psychology. The use of the EAS scale in research in the field of environmental psychology can provide significant information about people’s attitudes toward the environment. This is important for understanding how attitudes influence behaviors and decisions related to the environment. However, it is important to keep in mind that the conclusions are based on the specific findings of this study and further research and validation in different contexts and populations is needed to confirm the robustness and generalizability of the results.

## Data availability statement

The original contributions presented in the study are included in the article/Supplementary material, further inquiries can be directed to the corresponding author.

## Ethics statement

The studies involving humans were approved by the project was approved by a Research Ethics Committee (Registration Code: N°DR-0023-P-22). The studies were conducted in accordance with the local legislation and institutional requirements. The participants provided their written informed consent to participate in this study.

## Author contributions

AS-B was in charge of the project as the principal investigator. EP-F and AS-B participated in the study design. EP-F, AS-B, and GB-F collaborated in the survey design, data collection, and analysis. AS-B and JS wrote the first draft of the manuscript. All authors contributed to the article and approved the submitted version.
