# Supplementary material for: Evidence of validity and reliability of the environmental action scale in Peruvian university students
Source: Front Psychol. 2023 Nov 22;14:1232397. doi: 10.3389/fpsyg.2023.1232397 (PMC10702606; doi:10.3389/fpsyg.2023.1232397)
Supplement: Supplementary file 1 [file Table_1.DOCX]

| Appendix A. *Adaptación lingüística de la Escala de Acción Ambiental (EAS) /* *Linguistic adaptation of the Environmental Action Scale (EAS).* | | | |
| --- | --- | --- | --- |
|  | | | |
| N° | **Versión Original** (Alisat & Riemer, 2015) | **Versión Traducida** | **Versión Adaptada** |
| 1 | *Educated myself about environmental issues (e.g., through media, television, internet, blogs, etc.)* | Me informé sobre temas medioambientales (por ejemplo: a través de medios de comunicación, televisión, internet, blogs, etc.) | Se mantiene la traducción |
| 2 | *Participated in an educational event (e.g., workshop) related to the environment* | Participé en una actividad educativa (por ejemplo: un taller) relacionado con el medio ambiente | Se mantiene la traducción |
| 3 | *Organized an educational event (e.g., workshop) related to environmental issues.* | Organicé una actividad educativa (por ejemplo: un taller) relacionado con el medio ambiente | Se mantiene la traducción |
| 4 | *Talked with others about environmental issues (e.g., spouse, partner, parent(s), children, or friends).* | Hablé con otras personas sobre temas medioambientales (por ejemplo: esposo/a, pareja, padres, hijos, amigos, etc.) | Se mantiene la traducción |
| 5 | *Used online tools (e.g., YouTube, Facebook, Wikipedia, MySpace Blogs) to raise awareness about environmental issues.* | Usé herramientas en línea (por ejemplo: YouTube, Facebook, Wikipedia, Blogs de MySpace) para crear conciencia sobre temas medioambientales. | Usé herramientas en línea (p.ej., YouTube, Facebook, Instagram, Tik Tok) para crear conciencia sobre temas medioambientales (cuidado al medio ambiente, contaminación ambiental, etc.) |
| 6 | *Used traditional methods (e.g., letters to the editor, articles) to raise awareness about environmental issues.* | Usé métodos tradicionales (por ejemplo: cartas a un editor, artículos) para crear conciencia sobre temas medioambientales | Usé métodos tradicionales (p.ej., llamadas o correos a un editor, artículos o periódicos) para crear conciencia sobre temas medioambientales (cuidado al medio ambiente, contaminación ambiental, etc.) |
| 7 | *Personally wrote to or called a politician/government official about an environmental issue.* | Le escribí o llamé personalmente a un político o funcionario gubernamental sobre un tema medioambiental. | Se mantiene la traducción |
| 8 | *Became involved with an environmental group or political party (e.g., volunteer, summer job, etc.).* | Participé en un grupo o partido político ambientalista (por ejemplo: como voluntario, en un trabajo de verano, etc.) | Se mantiene la traducción |
| 9 | *Financially supported an environmental cause* | Apoyé financieramente una causa medioambiental | Se mantiene la traducción |
| 10 | *Took part in a protest/rally about an environmental issue.* | Participé en una protesta o manifestación sobre un tema medioambiental. | Se mantiene la traducción |
| 11 | *Organized an environmental protest/rally.* | Organicé una protesta o manifestación medioambiental. | Se mantiene la traducción |
| 12 | *Organized a boycott against a company engaging in environmentally harmful practices.* | Organicé un boicot contra una empresa que realizaba prácticas que dañan el medio ambiente. | Organicé un boicot (p.ej., campaña masiva para que las personas dejen de consumir o comprar un producto) contra una empresa que realizaba prácticas que dañan el medio ambiente |
| 13 | *Organized a petition (including online petitions) for an environmental cause.* | Organicé una petición (que incluyen las peticiones en línea) para una causa medioambiental | Organicé una petición (incluyendo las peticiones en línea) para una causa medioambiental (por ejemplo: reforestar bosques, impedir acciones mineras que dañan al medio ambiente, conseguir leyes para la protección de animales, etc.) |
| 14 | *Consciously made time to be able to work on environmental issues (e.g., working part time to allow time for environmental pursuits, working in an environmental job, or choosing environmental activities over other leisure activities).* | De manera consciente, dediqué tiempo para poder trabajar con temas medioambientales (por ejemplo: trabajar medio tiempo para tener tiempo para causas medioambientales, trabajar en un empleo medioambiental o elegir actividades medioambientales sobre otras actividades de esparcimiento) | Dediqué tiempo para poder trabajar en temas medioambientales (por ejemplo: trabajar parcialmente para tener tiempo y dedicarme a causas medioambientales, trabajar en un empleo medioambiental o elegir actividades medioambientales sobre otras actividades de esparcimiento) |
| 15 | *Participated in a community event which focused on environmental awareness.* | Participé en una actividad comunitaria que se enfocaba en la conciencia medioambiental. | Participé en una actividad comunitaria (limpieza de las calles, jardines públicos, parques, ríos, canales, playas, etc.) que se enfocaba en la conciencia medioambiental (para promover la importancia del cuidado al medio ambiente) |
| 16 | *Organized a community event which focused on environmental awareness.* | Organicé una actividad comunitaria que se enfocaba en la conciencia medioambiental. | Organicé una actividad comunitaria (por ejemplo: limpieza de las calles, jardines públicos, parques, ríos, canales, playas, etc.). |
| 17 | *Participated in nature conservation efforts (e.g., planting trees, restoration of waterways).* | Participé en iniciativas para conservar la naturaleza (por ejemplo: plantar árboles, restaurar canales) | Participé en iniciativas para conservar la naturaleza (por ejemplo: plantar árboles, restaurar canales, recoger la basura del río, etc.) |
| 18 | *Spent time working with a group/organization that deals with the connection of the environment to other societal issues such as justice or poverty.* | Pasé tiempo trabajando con un grupo u organización que se ocupa de la relación del medio ambiente con otros temas sociales tales como la justicia o la pobreza. | Se mantiene la traducción |
|  | | | |
